# Effects of microtrauma on joint structural changes in the Curdlan-injected SKG mouse model of ankylosing spondylitis

**DOI:** 10.1371/journal.pone.0360912

**Published:** 2026-10-15

**Authors:** Seung Hoon Lee, Dongju Kim, Chanhyeok Jeon, Hye-Ryeong Jo, Jeehee Youn, Sungsin Jo, Tae-Hwan Kim

**Affiliations:** 1 Hanyang University Institute for Rheumatology Research (HYIRR), Hanyang University, Seoul, South Korea; 2 Department of Anatomy & Cell Biology, College of Medicine, Hanyang University, Seoul, South Korea; 3 Department of Biology, College of Natural Sciences, Soonchunhyang University, Asan, Chungcheongnam-do, South Korea; 4 Department of Rheumatology, Hanyang University Hospital for Rheumatic Diseases, Seoul, South Korea; Ondokuz Mayıs Üniversitesi: Ondokuz Mayis Universitesi, TÜRKIYE

## Abstract

Although microtrauma is considered a potential environmental factor in the development of ankylosing spondylitis (AS), there is a lack of experimental research regarding its precise contribution to disease pathogenesis. To induce physiological microtrauma, the right ankles of SKG mice were immobilized for a week using surgical plaster and a pipette tip to stimulate compensatory mechanical overload in the contralateral limb. Following the removal of the immobilization, the mice were intraperitoneally injected with 3 mg of Curdlan. In this model, the non-immobilized left ankle served as the microtraumatized group, while the immobilized right ankle served as the internal control. Arthritis severity was clinically scored weekly for 5 weeks. At 6 weeks, ankle, hip, and sacroiliac (SI) joint changes were evaluated using histological and micro-CT analyses. The microtraumatized (left) ankles of Curdlan-injected SKG mice exhibited exacerbated arthritis severity, synovitis, and enthesitis compared with the control ankles. Micro-CT analysis revealed a significantly elevated inflamed bone volume in the microtraumatized ankles. Immunohistochemistry showed markedly higher expression of pro-inflammatory cytokines (TNF-α, IL-1β, and IL-6) in the synovium and enthesis of the microtraumatized ankles. Notably, distant pathological changes, including proteoglycan loss and joint space narrowing, were also observed in the left hip joints. Collectively, these findings indicate that compensatory microtrauma significantly exacerbates joint inflammation and pathological bone destruction in the Curdlan-injected SKG mouse model, highlighting mechanical stress as a critical aggravating factor in AS progression.

## Introduction

Ankylosing spondylitis (AS) is a type of chronic inflammatory disease in which inflammation occurs between vertebrae accompanied by arthritis, and enthesitis [1,2]. AS is likely to occur in up to 1–2% of HLA-B27-positive adults, indicating that environmental factors affect the development and progression of AS [3]. Environmental factors in AS include smoking, gut dysbiosis, infection, and microtrauma induced by mechanical loading [4]. These various environmental factors induce inflammation in various tissues and produce inflammatory cytokines such as tumor necrosis factor alpha (TNF-α) and interleukin-17 (IL-17) and promote the development and progression of AS [5]. However, despite many environmental factor studies on AS, literature regarding the important role of environmental factors in the development and progression of AS is lacking.

Microtrauma is a small injury caused by repeated mechanical stress on tissue characterized by a slow onset of symptoms [6]. Most microtrauma causes inflammation in the muscles, ligaments, and spine, and repeated microtrauma can lead to more severe conditions [7–9]. Mechanical stress that induces microtrauma mainly causes enthesitis through activation of TNF-α and IL-17/23 and affects new bone formation [10]. However, the relationship between mechanical stress and new bone formation remains uncertain. In response to mechanical stimulation such as microtrauma, skeletal muscle secretes various cytokines termed myokines [11], which may play a pivotal role in modulating joint inflammation and subsequent new bone formation.

The SKG mouse, a BALB/c strain carrying the *W163C* mutation in the *Zap70* gene, is a valuable model for autoimmune disease [12]. This mutation impairs thymic selection, resulting in the generation of numerous highly self-reactive T cells. Upon curdlan stimulation, these self-reactive T cells are activated and predominantly enrich proinflammatory cytokines such as TNF-α or IL-17A, which are therapeutic targets for human AS [13–16]. These pathological immune cells migrate to joints where they play a critical role in the initiation and progression of chronic inflammatory arthritis. Therefore, curdlan-injected SKG mice can serve as a relevant animal model for examining the effects of potential environmental factors on AS clinical features.

In the present study, the effects of microtrauma on AS were investigated using a curdlan-injected SKG mouse model. Microtrauma was found to exacerbate arthritis, enthesitis, and inflammation in the curdlan-injected SKG mouse model. Therefore, the role of microtrauma and inflammation in influencing the progression of AS was investigated in the present study.

## Materials and methods

### Experimental microtrauma model and assessment

All experimental procedures were performed in accordance with the Guide for the Care of Use of Laboratory Animal and approved by the Animals Ethics Screening Committee of Hanyang University (HY-IACUC-25-0034). Environmental enrichment was implemented to reduce stress and improve animal welfare. Nestlets were provided to facilitate nesting behavior, and mice were group-housed with same-sex conspecifics to maintain social interactions unless aggressive behavior was observed. Adequate amounts of bedding were supplied to promote physical activity and allow the expression of species-specific behaviors, including digging and burrowing. SKG mice were originally obtained from Dr. Sakaguchi (Osaka University, Japan) [12]. To immobilize the right ankle of 7-week-old SKG mice, a plastic stick was prepared from a 1,000-uL pipette tip by cutting it to retain approximately 2 cm of the upper portion. The plastic stick was positioned along the ankle and fixed in place using a medical adhesive bandage (MediPlex®, NEOMED PHARM Inc., Goyang-si, Korea). Cable ties (KR-CTS, Kalon Living, WA, Australia) were additionally used to prevent the plastic stick from falling out. Animals were monitored daily. A week later, all the devices were removed from the right ankle. The mice were subsequently divided randomly into two groups. One group was received PBS injection, whereas the other group received an intraperitoneally injected with 3 mg of curdlan (034–09901, FUJIFILM Wako Pure Chemical Corporation, Osaka, Japan) to induce arthritis in the AS mouse model. The mice were injected with the same amount of curdlan after 2 weeks [17–19]. Control mice were injected with PBS. All mice were monitored for up to 5 weeks and scored by the same observer, who was blinded. To quantitatively evaluate the severity of arthritis, the SKG mice ankles were scored using a previously described 5-point scoring system where 0 = no swelling or focal redness (normal), 1 = slight swelling and/or focal redness, 2 = low-to-moderate swelling, 3 = pronounced swelling with reduced paw function, and 4 = excessive swelling with deformity and joint rigidity [13]. Mice were sacrificed in a chamber using 30–70% CO_2_, and their ankle, femurs and SI joint were collected.

### Micro-CT analysis

Micro-CT analysis was performed as previously described [20]. The SI joints, femurs, and ankles of mice were fixed in 10% formalin (HT501640, Sigma-Aldrich, MO, USA) for a minimum of 1 day and analyzed using high-resolution micro-CT (Skyscan1272, Bruker micro-CT, Kontich, Belgium). All micro-CT data of mice were performed and analyzed with the 2nd analysis Lab (http://2ndanalysis.co.kr). The scanner was set at a voltage of 60 kV, an aluminum filter of 0.25 mm, and a resolution of 9 μm per pixel. The micro-CT image was used to perform three-dimensional (3D) histomorphometric analyses. The images were reconstructed with NRecon v1.7.3.1 software (Bruker micro-CT) +InstaRecon software (InstaRecon), analyzed with CTAn v1.20.3.0 software (Bruker micro-CT), and visualized using the 3D model visualization software CTVol v2.3.2.0 (Bruker micro-CT). For BMD analysis, the ROI was defined within the trabecular bone of the distal femur, excluding the cortical bone and growth -plate. For the assessment of ectopic new bone formation in the ankle, the ROI was defined around the entheseal region rather than the joint space, encompassing the peri-entheseal area where pathological bone formation was observed. The same anatomical landmarks and ROI dimensions were consistently applied to all animals across experimental groups.

### Histopathological analysis

The staining procedures were performed in accordance with standard protocols or following the manufacturer’s instructions. The SI joints and ankles of the sacrificed mice were fixed with 10% formalin for a week and decalcified with 10% formic acid (UN1779, Duksan Pure Chemicals, Ansan, Korea) at room temperature for a week. Paraffin-embedded tissue blocks were sectioned at 5-µm thickness. The slides were stained with hematoxylin (1.05174.0500, Merck, Darmstadt, Germany) and eosin (HT110216, Sigma-Aldrich) for histological evaluation, safranin O (ab150681, Abcam, Cambridge, UK) for cartilage analysis, and Masson’s trichrome (ab150686, Abcam) for total collagen. All slides were mounted with a permanent mounting medium (H-5000, Vector Laboratories, CA, USA).

To quantitatively evaluate the severity of synovitis, SKG mice ankles were scored using a previously described six-point scoring system where 0 = no pathological changes, 1 = minimal multifocal small (no measurable aggregates) subsynovial accumulation of inflammatory cells, 2 = diffuse small (no measurable aggregates) subsynovial accumulation of inflammatory cells, 3 = diffuse small subsynovial accumulation of inflammatory cells + 1–3 aggregates measuring at least 100 um in diameter, 4 = diffuse small subsynovial accumulation of inflammatory cells + > 3 aggregates measuring at least 100 μM in diameter, and 5 = diffuse sever inflammation with numerous aggregates [21]. To quantitatively evaluate the severity of enthesitis, SKG mice ankles were scored using a previously described four-point scoring system where 0 = no pain, 1 = mild tenderness, 2 = moderate tenderness, and 3 = wince or withdraw [22].

### Immunohistochemistry

Slides were stained with Dako REAL EnVision Detection system kit (K5007, DAKO, CA, USA) for immunostaining of TNF-α, IL-1β, IL-6, or MMP13. Paraffin-embedded slices were washed twice with Neo-clear (1.09843.500, Merck) and hydrated through a series of decreasing concentrations of ethanol (100% to 50%). The sections were incubated with 0.3% Triton X-100 (T8787, Sigma-Aldrich) in 1X PBS for 10 min and with BLOXALL (SP-6000, Vector Laboratories) for 10 min. Then, the slides were washed with 1X PBS and incubated with proteinase K (VB-6009, Vitro Vivo Biotech, MD, USA) for 10 min followed by incubation with 1:100 anti-TNF-ɑ (SC-52746, Santa Cruz Biotechnology, TX, USA), anti-IL-1β (12242, Cell Signaling Technology, MA, USA), anti-IL-6 (ab6672, Abcam), or anti-MMP13 (A16920, ABclonal, Seongnam-si, Korea) antibodies in antibody diluent (S3022, DAKO) at room temperature for 1 h. The sections were washed three times with 0.3% Tween-20 (8.22184.0500, Sigma-Aldrich) in 1X PBS, followed by incubation with Dako REAL Envision/HRP buffer. After 30 min, the slides were washed three times with 0.3% Tween-20 in 1X PBS and incubated with DAB-containing substrate working solution (Dako REAL substrate buffer containing with Dako REAL DAB+ Chromogen) for 1–5 min. The stained slides were washed with distilled water for 5 min, counterstained with hematoxylin for 1 min, and washed with distilled water for 15 min. For dehydration, the slides were immersed in a graded series of ethanol solution (50% to 100% ethanol), washed two times with Neo-clear, and mounted with permanent mounting medium (H-5000, Vector Laboratories). To quantify the expression of TNF-α, IL-1β and IL-6, the number of positively stained cells was determined among the total number of cells in ankle. High-power fields (HPF) were observed at 200 × magnification.

### Statistical analysis

Graph-Pad Prism 7.04 was used to produce graphs and statistical analyses. Statistical analysis was performed using the Mann-Whitney U test. The values are shown as the mean ± standard error of the mean (SEM). All experiments were performed at least three times. Asterisks represent the level of statistical significance (*p < 0.05, **p < 0.01, ***p < 0.001).

## Results

### Microtrauma exacerbates arthritis and inflamed bone formation in curdlan-injected SKG mice

The immobilization method on the right ankle was used to induce microtrauma through repeated stress on the left ankle. As shown in Fig 1A, twenty-three 7-week-old SKG mice were randomly divided into two groups. The right ankle was immobilized for 1 week, after which the mice received an intraperitoneal injection of either PBS or 3 mg of Curdlan. The same amount of PBS or curdlan was injected two weeks later. SKG mice with the right ankle fixed were observed to move only with the left ankle (S1 Fig in S1 File). Notably, the clinical severity of arthritis was significantly higher in the non-immobilized left ankle than in the immobilized right ankle in Curdlan-injected SKG mice (Fig 1B and C). Furthermore, micro-CT analysis revealed that inflamed bone volume was markedly increased in the non-immobilized left ankle compared to the immobilized right ankle (Fig 1C). Quantitative trabecular bone parameters were evaluated, while overall bone mineral density was found to remain unchanged (Fig 1D, S2 Fig and S1 Table in S1 File). In addition, as a control for the immobilization procedure, PBS- and curdlan-injected SKG mice that did not undergo unilateral immobilization showed no differences between the left and right hind ankles in inflammation-induced changes in paw thickness or inflamed bone formation (S3 Fig in S1 File). Taken together, these data demonstrate that compensatory microtrauma exacerbates arthritis and inflamed bone formation in Curdlan-injected SKG mice.

**Fig 1:**
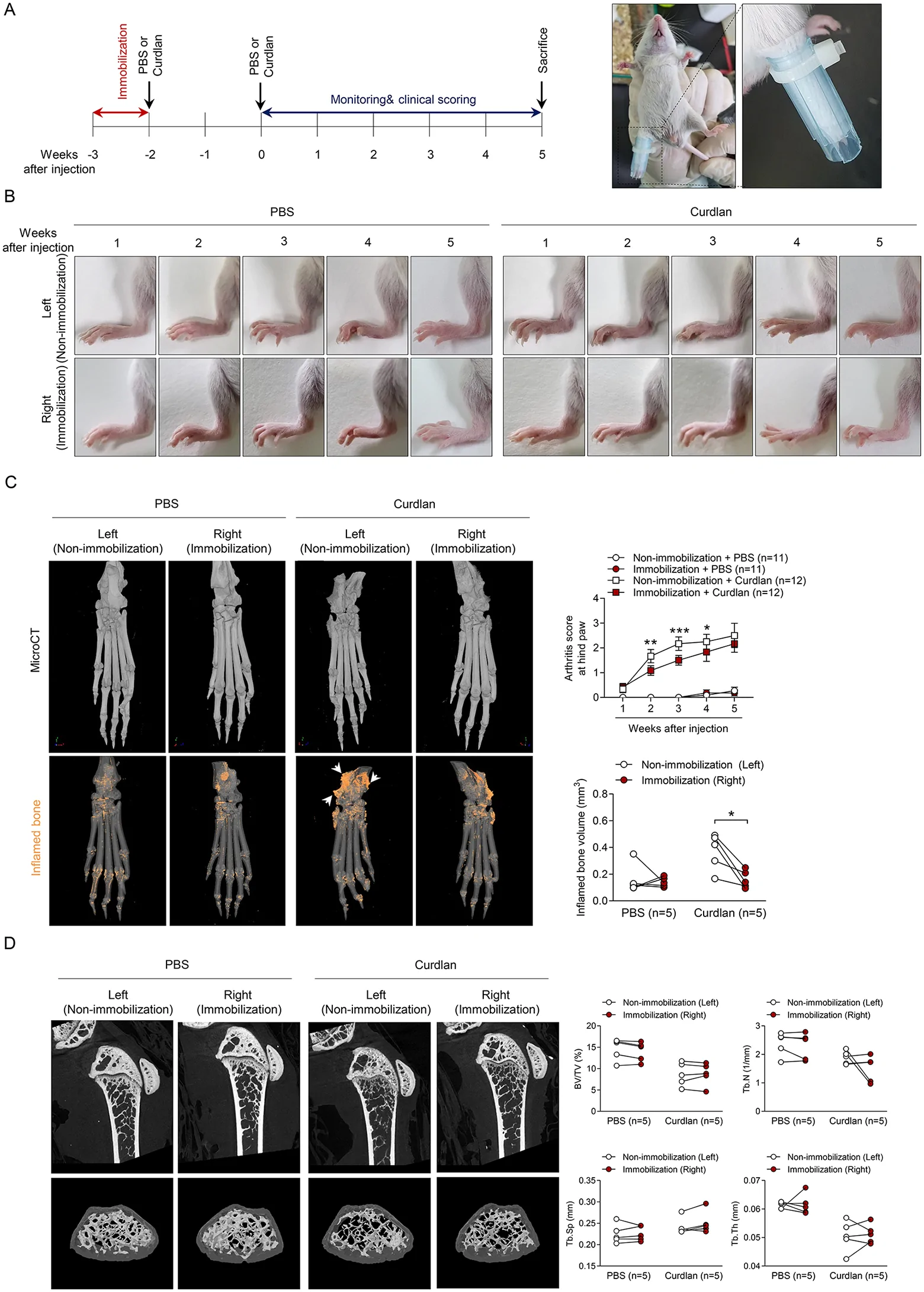
Increased inflamed bone formation in the microtraumatized ankle of Curdlan-injected SKG mice. (A) Experimental design. Right hind limb of 7-week-old SKG mice was immobilized for 1 week. (B) Representative gross images of both ankles during 5 weeks after curdlan injection. (C) Representative micro-CT images of ankles in each group. Orange color indicates inflamed ankle tissues. White arrow in micro-CT image indicates ectopic bone formation in the ankle. The arthritis scores are shown. Calculation of inflamed tissues and quantification data are shown. (D) Representative micro-CT images of the femurs in each group. Calculation of trabecular bone analysis and quantification data are shown. Data are expressed as the mean ± standard error of the mean (SEM). *p < 0.05, **p < 0.01, and ***p < 0.001 based on the Mann-Whitney U test.

### Microtrauma induces synovitis and enthesitis in curdlan-injected SKG mice

To evaluate the effect of microtrauma on joint pathology, comprehensive histological analyses and immunohistochemical staining were performed. Histological evaluation using H&E, Safranin O (SO), toluidine blue (TB), Sirius red, and Masson’s trichrome staining revealed that synovitis and enthesitis were substantially more pronounced in the non-immobilized left ankle than in the immobilized right ankle of Curdlan-injected SKG mice (Fig 2A and B). Furthermore, the number of cells positive for pro-inflammatory cytokines (TNF-α, IL-1β, and IL-6) was markedly increased in both the synovium and enthesis of the non-immobilized left ankle (Fig 2C and D; red arrows). Collectively, these findings indicate that microtrauma severely exacerbates local synovitis and enthesitis under systemic inflammatory conditions.

**Fig 2:**
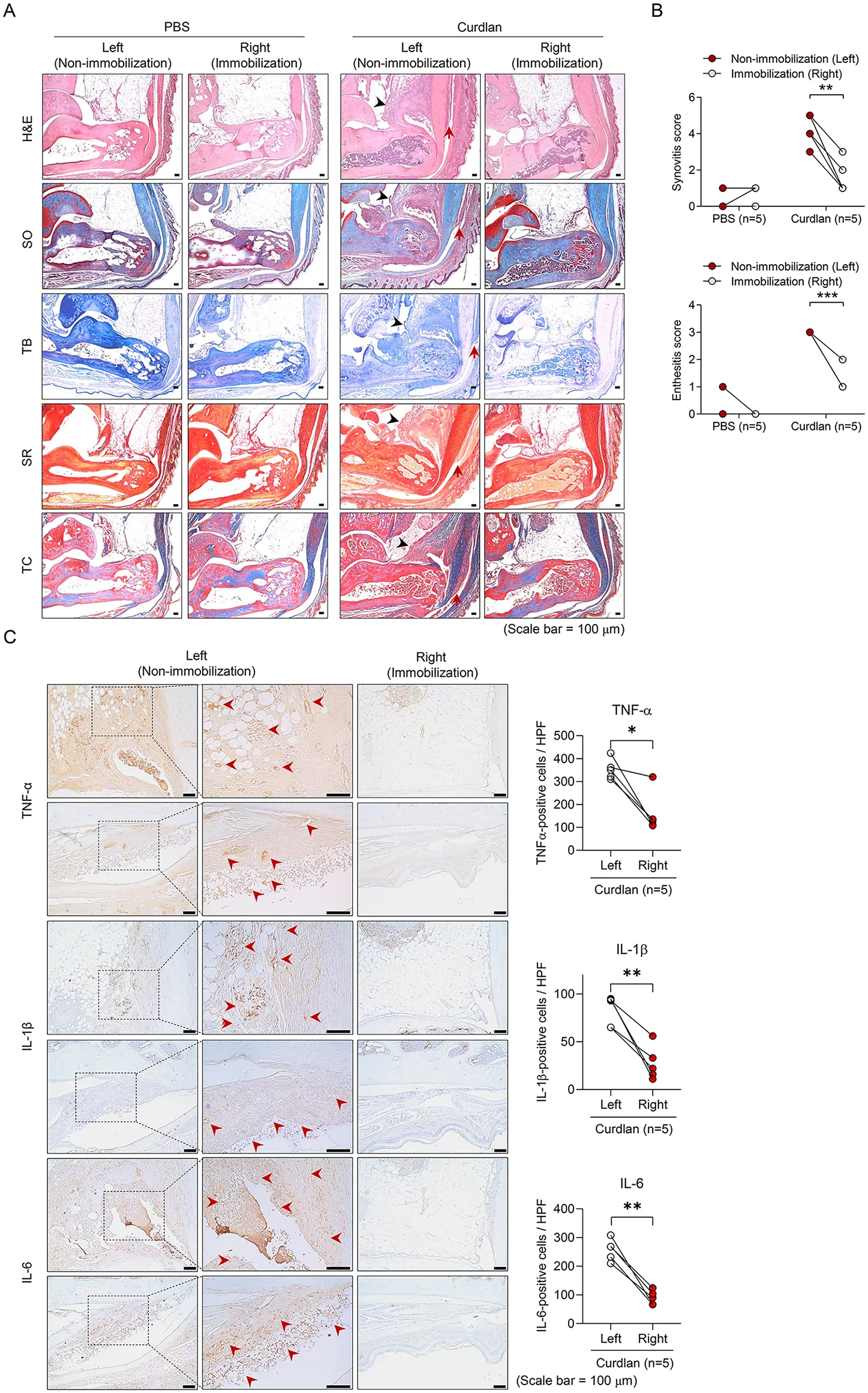
Increased severity of arthritis, synovitis, and enthesitis in the microtraumatized ankle of Curdlan-injected SKG mice. (A) Histological analysis using hematoxylin and eosin (H&E), safranin O (SO), and toluidine blue (TB) staining for cartilage, and Sirius red (SR) and trichrome (TC) for collagen. Black arrowhead indicates synovitis and red arrow enthesitis. (B) The synovitis score (upper) and enthesitis score (lower) are shown. (C) Immunohistochemical analysis of pro-inflammatory cytokines (TNF-α, IL-1β, and IL-6) in the ankle joints of immobilized versus non-immobilized legs in curdlan-treated SKG mice. (D) Cells positive for pro-inflammatory cytokines in ankles were counted. Red arrow indicates expression of pro-inflammatory cytokines. Data are expressed as the mean ± standard error of the mean (SEM). *p < 0.05, **p < 0.01 and ***p < 0.001 based on the Mann-Whitney U test.

### Microtrauma induces destruction of the SI joint in curdlan-injected SKG mice

Clinically, AS frequently involves the SI joint, leading to progressive structural destruction [23,24]. To determine whether distant mechanical stress from ankle microtrauma influences SI joint pathology, micro-CT and histological analyses were conducted. Micro-CT imaging revealed significant joint space narrowing specifically in the left SI joint of Curdlan-injected SKG mice (Fig 3A and B). This was corroborated by histological analyses using H&E, TB, and SO staining, which confirmed advanced joint destruction in the left SI joint (Fig 3C). In addition, the expression of matrix metallopeptidase 13 (MMP13), a key marker of cartilage degradation and joint destruction, was specifically upregulated in the left SI joint, suggesting a localized response driven by direct mechanical signal transduction (Fig 3D). Overall, these data indicate that altered biomechanics and subsequent microtrauma exacerbate the destruction of the distant SI joint in Curdlan-injected SKG mice.

**Fig 3:**
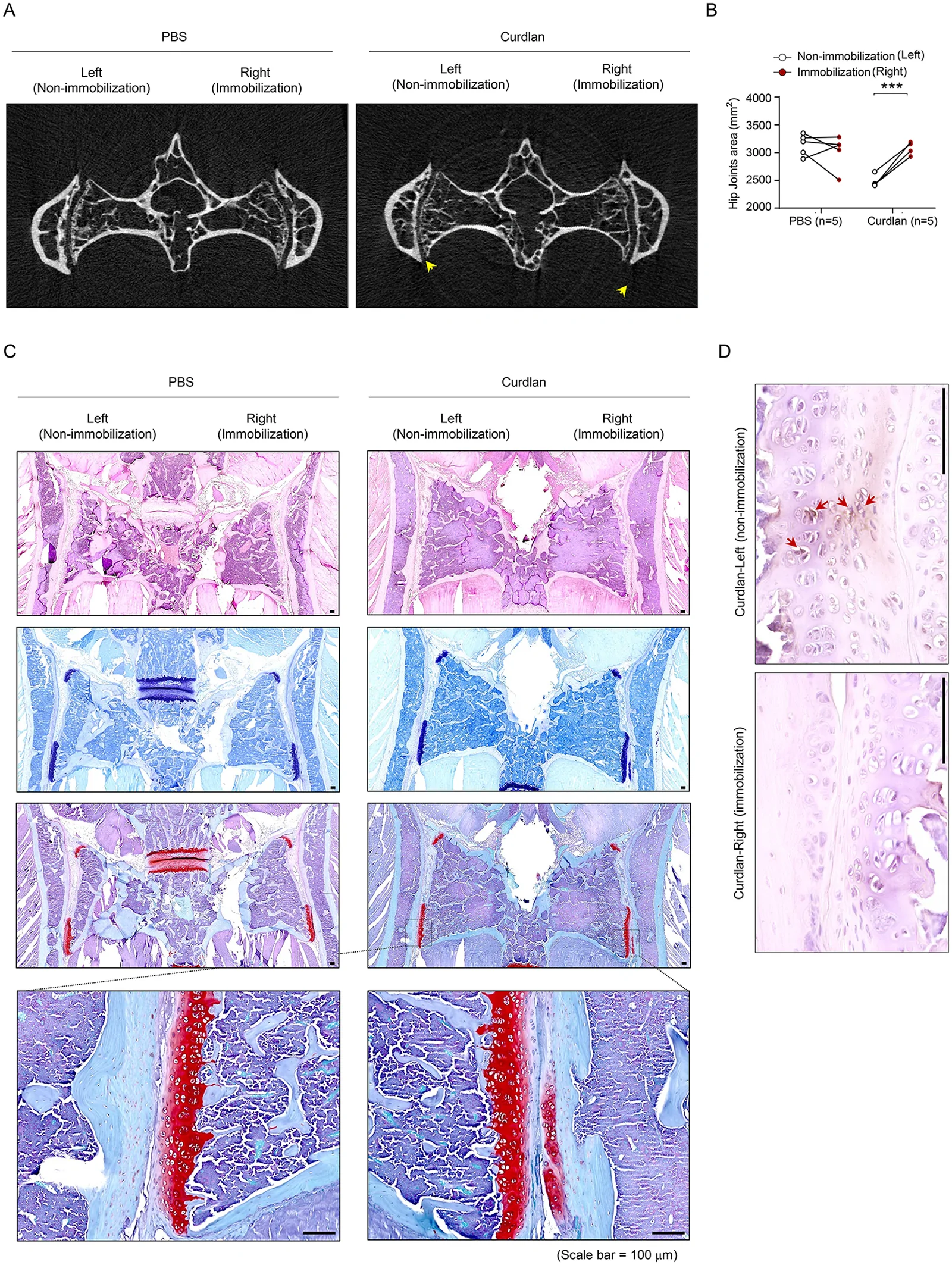
Structural changes and joint space narrowing of the SI joint in Curdlan-injected SKG mice with microtrauma. (A) Representative micro-CT images for the SI joint in each group. Yellow arrow indicates SI joint. (B) Calculation of hip joint area and quantification data are shown. (C) Histological analysis using hematoxylin and eosin (H&E; upper), toluidine blue (TB; middle), and safranin O (SO; lower) staining for cartilage. (D) Immunohistochemistry of MMP13 in the SI joint of non-immobilized and immobilized legs of curdlan-injected SKG mice. Data are expressed as the mean ± standard error of the mean (SEM). ***p < 0.001 based on the Mann-Whitney U test.

## Discussion

In the present study, the role of microtrauma on inflammation and new bone formation in the development and progression of AS were investigated using the unilateral hindlimb immobilization method applied to the right ankle. An increase in arthritis, enthesitis, and synovitis was observed in the left ankle of curdlan injected-SKG mice that received microtrauma. Notably, microtrauma increased the inflamed bone formation and expression of inflammatory cytokines (TNF-α, IL-1β, IL-6) in the ankle. In addition, a decrease in the hip spacing and SI joint that received microtrauma was observed. Furthermore, MMP13 expression increased in the SI joint that received microtrauma. Collectively, the results indicated that microtrauma at the immobilized ankle contributed to severe arthritis and enthesitis in the ankle of curdlan-injected SKG mice.

An important characteristic of AS is the onset of an outbreak in an adolescent male [25]. In previous studies, adolescent males were shown to frequently experience microtrauma and knee pain focused on the tibial tuberosity due to vigorous physical activity such as sports compared with females [26–28]. Repeated microtrauma wounds continue to scar the surrounding tissues, creating injuries in the muscles, ligaments, vertebrae, and discs. Notably, more than 50% of patients with AS reportedly experience trauma [29]. Furthermore, microtrauma was previously reported the cause of enthesitis, a symptom of AS [4]. Therefore, microtrauma may be an important initiator or mediator of AS development and progression.

Mechanical stress is a major contributor to the occurrence of microtrauma [30]. Sieper and Poddubnyy reported that mechanical stress accelerated heterotopic ossification of the cartilage and ligament surrounding these tissues [31]. The Wnt/β-catenin pathway was shown to play a crucial role in the formation of heterotopic ossification in AS [32,33]. In addition, mechanical stress stimulated heterotopic ossification via the MARK pathway [34]. Zhang et al. reported that when the mechanical stress was reduced using tail suspension, the heterotopic ossification decreased in the proteoglycan-induced AS animal model via the Wnt/b-catenin and MARK pathways [35].

Mechanical stress may also exert distinct and context-dependent effects on bone remodeling in AS. In an inflammatory environment, pro-inflammatory cytokines can promote osteoclast differentiation and activity, whereas repetitive mechanical stress, particularly at entheseal sites, may promote local osteogenic responses and pathological new bone formation [36,37]. Thus, bone resorption and pathological new bone formation are not necessarily mutually exclusive but may represent spatially and temporally distinct aspects of dysregulated bone remodeling in AS [36]. In the present study, a significant increase in pathological bone features in the non-immobilized left ankle was observed (Fig 1C). These findings suggest that the effects of mechanical loading in our model may be more prominent in local pathological bone formation at mechanically stressed sites than in generalized changes in bone mass. Nevertheless, the absence of detectable changes in BMD does not exclude alterations in local osteoclast or osteoblast activity. Further studies directly assessing osteoclast and osteoblast activity at mechanically stressed sites are needed to clarify the mechanisms underlying microtrauma-induced pathological bone formation.

Immobilization of one lower limb can induce microtrauma in the contralateral lower limb. Ikeda et al. reported a study that induced unilateral lower limb motion restriction through short-term cast fixation of one lower limb, which caused postural instability and postural control asymmetry, and confirmed that the center of pressure shifts from the fixed side to the unfixed side [38]. Basciani et al. reported that imbalance of the center of gravity causes microdamage [39]. Additionally, Shedge et al. reported that patients with lateral ankle sprain and imbalance of the center of gravity develop sacroiliac joint dysfunction [40]. These observations support the possibility that altered biomechanics following unilateral immobilization may influence joints distant from the immobilized site.

However, changes at non-immobilized sites should also be interpreted in the context of broader alterations in physical activity and gait biomechanics. Unilateral joint immobilization or restriction can alter walking speed and gait mechanics and induce compensatory changes in mechanical loading at non-immobilized and contralateral sites [41,42]. Therefore, some pathological changes observed in the non-immobilized ankle and SI joint in our study may reflect not only increased local compensatory loading but also redistributed mechanical stress resulting from altered gait and overall locomotor activity. Future studies incorporating quantitative assessments of locomotor activity, gait biomechanics, and site-specific mechanical loading will be necessary to distinguish these effects. Although body weight did not significantly during immobilization (S4 Fig in S1 File), we did not directly quantify locomotor activity or gait biomechanics in our study. Future studies incorporating quantitative assessments of locomotor activity, gait biomechanics, and site-specific mechanical loading will therefore be necessary to distinguish systemic biomechanical effects from local responses.

Mechanical stress and microtrauma may contribute to inflammation not only through direct tissue damage but also by altering the local immune microenvironment. Tissue injury can promote the release of inflammatory mediators and facilitate the recruitment and activation of immune cells, including macrophages, thereby amplifying local inflammatory responses. Macrophage polarization is increasingly recognized as an important component of the inflammatory joint microenvironment. Recent integrative analyses of osteoarthritis synovial tissues have identified macrophage polarization-related molecular signatures (such as MYC, SIK1, NFIL3) associated with immune cell infiltration, further supporting a close relationship between synovial inflammation and macrophage phenotypes [43]. In this context, the increased expression of TNF-α, IL-1β, and IL-6 observed following microtrauma in our study may reflect, at least in part, alterations in the local immune microenvironment induced by biomechanical stress. However, because macrophage infiltration and polarization were not directly evaluated in the present study, further studies are required to clarify the cellular mechanisms linking microtrauma to local inflammation.

Biological responses to physical and environmental stress may involve interconnected signaling networks rather than a single molecular pathway. Recent network-based studies of joint pathology have identified multiple interacting molecular targets and inflammatory signaling pathways associated with joint damage, including estrogen, thyroid hormone, ErbB, MAPK, PI3K-Akt, and mTOR signaling pathways [44,45]. Although these studies focused on osteoarthritis, they suggest that microtrauma-induced joint pathology in AS may involve coordinated interactions among mechanotransduction, inflammatory signaling, and tissue-remodeling pathways.

Numerous reports exist on the effects of FGF16 on proliferation of cardiomyocytes in heart development; however, the inflammatory effects of FGF16 are relatively less known. FGFs contained in the same FGF subfamily are structurally similar and act by binding to the same FGF receptor. Recently, Hung et al. reported the FGF9 subfamily such as FGF16, induces the hypertrophy of chondrocyte, skeletal angiogenesis, and the formation of mineralized bone formation [46]. In addition, Deng et al. reported that FGF9 signaling induces inflammation and neuronal apoptosis by affecting the recruitment of M1-associated macrophages, IL-1β expression, and ERK pathway signaling [47]. In the present study, arthritis and expression of pro-inflammatory cytokines (TNF-α, IL-1β, and IL-6) increased more in the non-immobilized left ankle than in the immobilized right ankle of curdlan-injected SKG mice (Fig 1 and 2). RNA sequencing analysis of muscle cells exposed to shear stress showed FGF16 expression was significantly increased compared with other FGF genes (S5A Fig in S1 File). FGF16 expression was also increased in the muscle of the left femur and ankle that received microtrauma in curdlan-injected SKG mice (S5B and S5C Fig in S1 File). These results suggest the possibility that FGF16 may contribute to inflammation and inflammatory bone formation in a curdlan-injected SKG mice with experimental microtrauma. However, the effects of FGF16 on AS in *in vitro* models need further investigation.

The present study had several limitations. First, data on overexpression and blocking of FGF16 *in vivo* and *in vitro* were not available. Second, ankylosis of the SI joint was induced in the non-immobilized left SI joint in curdlan-injected SKG mice. However, which microtrauma can induce ankylosis of the SI joint remains unclear. Third, although microtrauma increased both inflammation and intramuscular FGF16 expression, the mechanism underlying the upregulation of FGF16 remains unclear. Another limitation of the present study is that the effects of FGF16 on immune cells were not investigated. To evaluate the role of FGF16 in inflammation and bone metabolism, we examined its effects on LPS-stimulated macrophages, osteoblasts, and osteoclasts; however, no significant changes were observed in any of these cell types (data not shown). Given the pivotal roles of Th1 and Th17 cells in inflammatory responses and inflammatory bone diseases, the possibility that FGF16 influences these immune cell populations cannot be excluded. Therefore, further studies are warranted to elucidate whether FGF16 modulates immune cell function and contributes to inflammation through immune-mediated mechanisms. Fourth, the mice used in the experimental microtrauma model were 5 mice per group (repeated independently two times). This may reduce the reliability of the conclusion due to the small sample size, and this part should be considered when confirming the effect of joint destruction and inflammation due to microtrauma.

## Conclusion

In conclusion, the frequency and severity of arthritis, synovitis, and enthesitis were significantly increased in the microtraumatized ankle. Furthermore, structural destruction and pathological bone formation were exacerbated in the corresponding SI joint. These findings strongly indicate that compensatory microtrauma, induced by unilateral ankle immobilization, acts as a critical aggravating factor that contributes to the severity of arthritis, synovitis, enthesitis, and distant SI joint pathology in Curdlan-injected SKG mice.

## Supporting information

S1 File Supplementary figures and table. This file contains the following supplementary figures and table: S1 Fig, The mouse model of microtrauma using hindlimb immobilization; S2 Fig, Effects of microtrauma on cortical bone properties in ankles of SKG mice; S3 Fig, The change of hind paw thickness and inflamed bone volume in bilateral ankles of curdlan-injected SKG mice; S4 Fig, Effect of the unilateral immobilization procedure on body weight in SKG mice; S5 Fig, FGF16 is highly expressed in skeletal muscle with microtrauma; S1 Table, Trabecular bone parameters.
(DOCX)

S1 Data Raw Data. (ZIP)

## Abbreviations

AS: ankylosing spondylitis
SI: sacroiliac
TNF-α: tumor necrosis factor alpha
IL-1β: interleukin 1β
IL-6: interleukin 6
IL-17: interleukin-17
SKG mice: ZAP-70 mutant SKG mice
Micro-CT: micro-computed tomography
H&E: hematoxylin & eosin
SO: safranin O
TB: toluidine blue
SR: sirius red
TC: trichrome
MMP13: matrix metallopeptidase 13
FGF16: fibroblast growth factor 16
