# Supplementary material for: Effects of microtrauma on joint structural changes in the Curdlan-injected SKG mouse model of ankylosing spondylitis
Source: PLoS One. 2026 Oct 15;21(10):e0360912. doi: 10.1371/journal.pone.0360912 (PMC13671563; doi:10.1371/journal.pone.0360912)
Supplement: Supplementary file 1 [file pone.0360912.s001.docx]

**S1 Fig. The mouse model of microtrauma using hindlimb immobilization.**

Representative images of SKG mice during immobilization of the right ankle. The mice were allowed to move on its forelimbs and left hindlimbs.

**S2 Fig. Effects of microtrauma on cortical bone properties in ankles of SKG mice.**

Cortical bone volume (Ct.TV) and cortical thickness (CT.Th) were measured using micro-CT. Data are expressed as the mean ± standard error of the mean (SEM).

**S3 Fig. The change of hind paw thickness and inflamed bone volume in bilateral ankles of curdlan-injected SKG mice.**

(A) Hind paw thickness was quantified. (B) Calculation of inflamed tissues and quantification data are shown. Data are expressed as the mean ± standard error of the mean (SEM).

**S4 Fig. Effect of the unilateral immobilization procedure on body weight in SKG mice.**

Body weight was measured daily for 7 days following the immobilization procedure. The graph shows a comparison between the non-immobilized control group (black circles) and the immobilized group (red squares). Data are expressed as the mean ± standard error of the mean (SEM).

**S5 Fig. FGF16 is highly expressed in skeletal muscle with microtrauma.**

(A) RNA sequencing of muscle cells subjected to shear stress. (B) Immunohistochemistry of FGF16 in the ankles and femur and tibia muscles in non-immobilized and immobilized legs of curdlan-injected SKG mice. FGF16 mRNA levels in the femur and tibia muscles were measured using real-time PCR (n = 3). Data are expressed as the mean ± standard error of the mean (SEM). ****p* < 0.001 based on two-tailed Student’s *t*-test.

**S1 Table. Trabecular bone parameters (Mean ± SEM)**

|  | PBS | | Curdlan | |
| --- | --- | --- | --- | --- |
|  | Left  (Non-immobilization) | Right  (Immobilization) | Left  (Non-immobilization) | Right  (Immobilization) |
| BT/TV (%) | 14.60 ± 1.14 | 14.0. ± 1.01 | 8.68 ± 1.22 | 8.73 ± 1.16 |
| Tb.N (1/mm) | 2.38 ± 0.19 | 2.30 ± 0.21 | 1.70 ± 0.19 | 1.69 ± 0.20 |
| Tb.Sp (mm) | 0.026 ± 0.0004 | 0.026 ± 0.0013 | 0.026 ± 0.0018 | 0.025 ± 0.0006 |
| Tb.Th (mm) | 0.061 ± 0.0005 | 0.062 ± 0.0016 | 0.051 ± 0.0024 | 0.051 ± 0.0015 |
