# Supplementary material for: Effects of microtrauma on joint structural changes in the Curdlan-injected SKG mouse model of ankylosing spondylitis
Source: PLoS One. 2026 Oct 15;21(10):e0360912. doi: 10.1371/journal.pone.0360912 (PMC13671563; doi:10.1371/journal.pone.0360912)
Supplement: Supplementary file 2 [file pone.0360912.s004.docx]

**Response to reviewer #1 comments**

(indicated in blue)

Reviewer #1: In general, it is a good written manuscript. The manuscript presented in clear and comprehensible fashion and written in standard English. The statistical analysis has been performed appropriately.

Response: We are very grateful for the reviewer’s positive comments and constructive suggestions, which have significantly improved the impact of our study. We are particularly encouraged by the reviewer’s enthusiasm for our work.

**Response to reviewer #2 comments**

(indicated in blue)

Reviewer #2: The study addresses an interesting and relevant question regarding the role of microtrauma in ankylosing spondylitis using the SKG mouse model, and the experimental approach combining immobilization, curdlan induction, histology, and micro-CT is appropriate. However, there are several important scientific, methodological, and interpretational issues that need to be addressed before the work can be considered for publication.

Response: We are very grateful for the reviewer’s positive comments and constructive suggestions, which have significantly improved the impact of our study. We are particularly encouraged by the reviewer’s enthusiasm for our work. In response to the specific points raised, we have substantially revised the manuscript. Our point-by-point responses to the reviewer’s comments are provided below, with all changes in the revised manuscript marked in Blue for clarity. All changes to the manuscript are highlighted in Yellow.

1. The most critical issue is the conceptual inconsistency in how microtrauma is defined and modeled. The study claims that immobilization of the right ankle induces microtrauma, yet the main effects are observed in the contralateral non-immobilized left ankle, which is interpreted as being subjected to mechanical overload. This creates confusion about whether the model is actually testing immobilization-induced effects or compensatory overuse. The authors need to clearly define what constitutes microtrauma in their model and justify why the contralateral limb is the primary site of pathology. Without this clarification, the central premise of the study remains ambiguous.

Response: We appreciate the reviewer’s insightful comment and agree that the conceptual basis of our microtrauma model required clearer articulation. While the right ankle was immobilized, the primary intention of this model was to induce compensatory mechanical overload and subsequent microtrauma in the contralateral non-immobilized (left) ankle. To support our conceptual basis, Ikeda et al. (2023) demonstrated that short-term cast immobilization of a unilateral lower limb causes postural instability and asymmetry, shifting the center of pressure from the immobilized side to the unfixed side. Furthermore, Basciani et al. (2024) noted that such an imbalance in the center of gravity can lead to microdamage. Based on these previous findings, our unilateral immobilization model was utilized to intentionally induce compensatory microtrauma in the contralateral limb.

We apologize for the initial ambiguity. We have now explicitly defined this mechanism and justified our model in the revised Discussion section (Lines 238–248).

-Reference-

1. Ikeda T, Oka S, Tokuhiro J, Suzuki A, Matsuda K. Short-Term Cast Immobilization of a Unilateral Lower Extremity and Physical Inactivity Induce Postural Instability during Standing in Healthy Young Men. Healthcare (Basel). 2023 Sep 13;11(18):2525. doi: 10.3390/healthcare11182525.

2. Basciani S, Zampogna B, Gregori P, Shanmugasundaram S, Guelfi M, Marinozzi A. Current concepts in ankle microinstability and ankle functional instability. J Clin Orthop Trauma. 2024 Mar 12;51:102380.

2. Another major issue is the lack of mechanistic depth. While the manuscript reports increased inflammatory cytokines and mentions FGF16 as a potential mediator, there is no functional validation. The authors themselves acknowledge that there are no in vivo or in vitro experiments manipulating FGF16 expression or activity. As a result, the mechanistic conclusions are speculative and not sufficiently supported by the data. Additional experiments or a more cautious interpretation are required.

Response: We sincerely appreciate the reviewer’s critical feedback regarding the mechanistic depth of our study. We agree that the exact functional role of FGF16 lacks direct validation in our current model, and as the reviewer rightly pointed out, our conclusions should reflect a more cautious interpretation.

To address this concern, we performed comprehensive in vitro assays to investigate the direct functional role of FGF16. However, we did not observe any significant phenotypic changes following FGF16 treatment (10, 25, and 50 ng/mL). Specifically, FGF16 did not affect the cellular proliferation of RAW264.7 cells (Author Response Image 1), nor did it alter the mRNA expression of pro-inflammatory markers (Il1b, Il6, Tnfa, Cox2, iNos, and Ho1) in LPS-stimulated RAW264.7 macrophages (Author Response Image 2). Furthermore, FGF16 treatment showed no direct impact on bone remodeling processes, as it failed to influence RANKL-induced osteoclast differentiation (Author Response Image 3) and osteoblast differentiation (Author Response Image 4).

Because these experiments yielded negative results, we provide these data exclusively in this response letter for the reviewer's reference and have opted not to include them in the revised manuscript.

This lack of direct response suggests that FGF16 may not act directly on these specific cells, but rather might exert its biological functions by modulating adaptive immune responses, such as Th1 and Th17 cell populations, which are key drivers in inflammatory diseases. Because we did not evaluate these specific immune cell subsets in the current study, we cannot make definitive mechanistic claims. Following your valuable suggestion, we have toned down our speculative conclusions, explicitly acknowledged this limitation, and incorporated the need for future studies on adaptive immune cell subsets into the revised Discussion section (Lines 269–282).

3. The experimental design also raises concerns. The use of the contralateral limb as a control is problematic because it is not a true control; it is subjected to altered biomechanics due to immobilization of the opposite limb. A proper control group without immobilization or with balanced loading is needed to isolate the effect of microtrauma. Additionally, the sample size appears limited, and there is insufficient detail about power calculation or variability, which affects the robustness of the conclusions.

Response: We appreciate the reviewer's thoughtful comments on our experimental design. We agree that using the contralateral limb as a control presents limitations due to altered biomechanics. To isolate the effect of microtrauma, we evaluated a true control group treated with PBS and Curdlan without immobilization. In this group, we found no significant differences in ankle thickness or inflamed bone volume between the left and right ankles. This confirms that Curdlan induces equal inflammation bilaterally, and we have updated the revised manuscript (Lines 182–185) and Supplementary Figure 3 to reflect this (Please refer to S3 Fig). Regarding the sample size, we based our experimental design of n=5 per group on recent established studies using the Curdlan-injected SKG mouse model (Nakamura et al., 2024; Whangbo et al., 2025; Ke et al., 2026). We originally conducted two independent replicates of the experiment; however, considerable variability between the two batches prevented us from pooling the data for analysis. We fully accept the reviewer's point that this limited sample size may reduce the robustness of our conclusions. We have now explicitly added this to the Limitations section of the manuscript and will strongly consider increasing the sample size in future studies.

-Reference-

Nakamura A, Jo S, Nakamura S, Aparnathi MK, Boroojeni SF, Korshko M, Park YS, Gupta H, Vijayan S, Rockel JS, Kapoor M, Jurisica I, Kim TH, Haroon N. HIF-1α and MIF enhance neutrophil-driven type 3 immunity and chondrogenesis in a murine spondyloarthritis model. Cell Mol Immunol. 2024 Jul;21(7):770-786. doi: 10.1038/s41423-024-01183-5.

Whangbo M, Ko E, Kim D, Jeon C, Jo HR, Lee SH, Youn J, Jo S, Kim TH. Wnt5a exacerbates pathological bone features and trabecular bone loss in curdlan-injected SKG mice via osteoclast activation. BMB Rep. 2025 Feb;58(2):75-81. doi: 10.5483/BMBRep.2024-0155.

Ke D, Dai H, Su Y, Zhu H, Liu X, Bai X, Zhang C, Xu J, Zhang J. Single-immunocyte transcriptomics reveal the role of natural killer cell-dependent exogenous antigen presentation in ankylosing spondylitis severity. Exp Mol Med. 2026 Feb;58(1):254-271. doi: 10.1038/s12276-025-01619-6.

4. There are also issues with data interpretation. The manuscript claims systemic effects, such as changes in the sacroiliac joint and hip, but does not convincingly demonstrate how localized ankle microtrauma leads to distant joint pathology. The causal link is not clearly established, and alternative explanations such as systemic inflammation induced by curdlan are not adequately discussed.

We appreciate the reviewer's critical evaluation of our data interpretation. We agree that the manuscript needed to better explain how localized ankle immobilization leads to distant SI joint pathology, and to address the alternative explanation of Curdlan-induced systemic inflammation. The link between ankle pathology and the SI joint can be explained by altered global biomechanics. Ikeda et al. (2023) showed that unilateral lower limb fixation shifts the center of pressure to the unfixed side, causing postural control asymmetry. Similarly, Shedge et al. (2024) observed that lateral ankle sprains lead to SI joint dysfunction. Therefore, the distant joint pathology in our model is driven by a biomechanical chain reaction that causes compensatory microtrauma. Furthermore, we can exclude Curdlan-induced systemic inflammation as the sole cause of these distant effects. As shown in Figure 3, there was no significant difference in the SI joint of the immobilized side between the PBS and Curdlan groups. This confirms that the observed SI joint pathology is specifically dependent on the mechanical microtrauma induced by our model, rather than just systemic inflammation. We have added these clarifications to the revised manuscript (please refer to lines 238–248).

-Reference-

Ikeda T, Oka S, Tokuhiro J, Suzuki A, Matsuda K. Short-Term Cast Immobilization of a Unilateral Lower Extremity and Physical Inactivity Induce Postural Instability during Standing in Healthy Young Men. Healthcare (Basel). 2023 Sep 13;11(18):2525. doi: 10.3390/healthcare11182525.

Shedge SS, Ramteke SU, Samal S. Integrated Rehabilitation Approach Utilizing Swiss Ball Training, Mulligan Taping, and Mobilization With Movement for Simultaneous Management of Sacroiliac Joint Dysfunction and Lateral Ankle Sprain in a Badminton Athlete: A Case Study. Cureus. 2024 Mar 26;16(3):e56942. doi: 10.7759/cureus.56942.

5. The statistical analysis is relatively simple and may not be sufficient for the complexity of the data. Only the Mann-Whitney U test is used, and there is no indication of correction for multiple comparisons despite multiple endpoints being analyzed. This raises concerns about the reliability of the reported significance.

Response: We appreciate the reviewer’s careful evaluation of our statistical methodology. We understand the concern regarding the use of the Mann-Whitney U test and the lack of multiple comparison corrections. We would like to clarify the rationale behind our statistical approach. In our experimental model, the physiological effects of microtrauma were not observed in the PBS-treated (non-inflammatory) group. The primary hypothesis for these specific endpoints was to evaluate the pathological exacerbation caused by microtrauma exclusively within the inflammatory context (the Curdlan-treated group). Therefore, rather than conducting an exploratory multi-group comparison across all conditions, our statistical analysis was pre-defined to perform a focused, direct two-group comparison within the Curdlan-treated cohort. Because we specifically conducted independent pairwise comparisons (comparing two specific groups) rather than multiple simultaneous comparisons across several groups, the Mann-Whitney U test was statistically appropriate, and correction for multiple comparisons was not strictly required. We have added a clearer explanation of this statistical rationale to the Statistical Analysis section in the revised manuscript.

6. The methods section lacks some important details. For example, more information is needed on randomization procedures, blinding beyond scoring, reproducibility of the immobilization technique, and how consistency of microtrauma induction was ensured across animals. These details are essential for reproducibility.

Response: We appreciate the reviewer's valuable feedback. We strongly agree that these methodological details are crucial for experimental reproducibility. In accordance with your suggestions, we have expanded the Methods section to explicitly describe the randomization procedures, the blinding methods utilized beyond outcome scoring, the reproducibility of the immobilization technique, and the protocols used to ensure consistent microtrauma induction across the subjects. Please refer to lines 88–99 in the revised manuscript for these updated descriptions. Additionally, to help with your understanding of the procedure, we have attached an illustrative figure below, so please kindly refer to it.

7. The discussion section overstates the conclusions in several places. The manuscript suggests that microtrauma is a key driver of ankylosing spondylitis pathogenesis, but the data only support an exacerbating effect in a specific animal model under artificial conditions. The authors should tone down these claims and better align conclusions with the experimental evidence.

Response: We appreciate the reviewer's valuable comment. We agree with the concern that our initial conclusion regarding the role of microtrauma in ankylosing spondylitis pathogenesis was overstated. Following the reviewer's recommendation, we have carefully moderated our claims to better align with our experimental data. We have revised the text to remove the implication that microtrauma is a major driving cause of the disease. Instead, we now clearly conclude that microtrauma significantly exacerbates disease symptoms in the Curdlan-injected SKG mouse model under our experimental conditions (Please refer to lines 262–264 in the revised manuscript).

We sincerely appreciate the reviewer’s constructive feedback advising us to moderate the tone of our conclusions. Therefore, we have toned down the language throughout the manuscript to avoid over-interpretation and these changes have been highlighted in the revised text. As part of this effort, we have revised the title of the manuscript from "Microtrauma exacerbates joint destruction..." to "Effects of microtrauma on joint structural changes in the curdlan-injected SKG mouse model of ankylosing spondylitis" to provide a more objective and scientifically rigorous description of our findings.

8. Finally, there are minor clarity and presentation issues, including wording inconsistencies, occasional grammatical errors, and the need for clearer figure descriptions, especially regarding how microtrauma is visually represented and quantified in the figures.

Response: We sincerely appreciate the reviewer for carefully pointing out the typos, grammatical errors, and presentation issues in the manuscript. We have re-reviewed the entire manuscript and corrected all grammatical errors and wording inconsistencies. Additionally, we have corrected typos and revised the descriptions for Fig. 1C, Fig. 1D, and Fig. 2B to provide a clearer explanation of how microtrauma is visually represented and quantified.

**Response to reviewer #3 comments**

(indicated in blue)

Reviewer #3: The manuscript addresses a clinically relevant topic by investigating the role of microtrauma (mechanical stress) in the pathogenesis of Ankylosing Spondylitis (AS). Using the curdlan-injected SKG mouse model, the authors ingeniously employed a unilateral immobilization method to induce compensatory stress, observing exacerbated inflammation and joint destruction. While the study provides interesting insights into the potential involvement of FGF16, the current version exhibits significant gaps in experimental logic, the presentation of core data (particularly regarding FGF16), and the biophysical explanation of systemic stress distribution. Major revisions are required to strengthen the validity of the conclusions before further consideration for publication.

Response: We are very grateful for the reviewer’s positive comments and constructive suggestions, which have significantly improved the impact of our study. We are particularly encouraged by the reviewer’s enthusiasm for our work. In response to the specific points raised, we have substantially revised the manuscript. Our point-by-point responses to the reviewer’s comments are provided below, with all changes in the revised manuscript marked in Blue for clarity. All changes to the manuscript are highlighted in Yellow.

1. The study uses the contralateral ankle of the same mouse as a control. The authors must clarify whether the compensatory stress generated by "fixing the right ankle" is sufficient and appropriate to represent "microtrauma" under daily physiological conditions. Please provide the scientific rationale for selecting this unilateral immobilization model.

Response: We appreciate the reviewer’s insightful comment and agree that the scientific rationale for using the unilateral immobilization model to represent microtrauma requires further clarification. Our primary objective in immobilizing the right ankle was to intentionally induce compensatory mechanical overload in the contralateral, non-immobilized (left) ankle during the mice's daily ambulation. To support this physiological rationale, Ikeda et al. (2023) demonstrated that short-term unilateral lower extremity cast immobilization causes postural instability and control asymmetry, significantly shifting the center of pressure from the fixed side to the unfixed, weight-bearing side. In our model, this forced shift requires the contralateral limb to constantly bear excess mechanical stress during normal cage activities. We believe this continuous, repetitive overload effectively and appropriately simulates the physiological conditions of microtrauma. Based on this strong rationale, we selected the unilateral immobilization model. We have explicitly detailed this scientific justification in the revised manuscript (Please refer to lines 88-99, 238–248).

Additionally, to help with your understanding of the procedure, we have attached an illustrative figure below, so please kindly refer to it.

-Reference-

Ikeda T, Oka S, Tokuhiro J, Suzuki A, Matsuda K. Short-Term Cast Immobilization of a Unilateral Lower Extremity and Physical Inactivity Induce Postural Instability during Standing in Healthy Young Men. Healthcare (Basel). 2023 Sep 13;11(18):2525. doi: 10.3390/healthcare11182525.

2. The use of surgical plaster and pipette tips for immobilization may significantly limit the mobility of the mice and induce systemic stress. The authors should provide supplementary data on body weight changes or food intake during the immobilization period to rule out the interference of systemic stress on inflammatory levels.

Response: We thank the reviewer for this valuable suggestion. We agree that it is crucial to rule out the interference of systemic stress. As requested, we measured the daily body weight changes of the mice during the immobilization period to confirm whether the immobilization procedure caused systemic stress. We confirmed that there was no significant weight change due to immobilization, which we have now included below. During this period, food pellets were deliberately placed on the cage floor to ensure the immobilized mice had easy access to nutrition and to prevent feeding-related stress. Because the food was mixed with the bedding and scattered, accurate quantification of food intake throughout the immobilization period was unfortunately not possible. However, the lack of body weight loss indirectly confirms that the mice fed adequately and did not suffer from severe systemic stress. Please refer to lines 245-248 in the revised manuscript.

3. The results suggest that increased weight-bearing on the left ankle led to destruction in the left sacroiliac (SI) joint. Please explicitly discuss the mechanism by which mechanical stress from the ankle is transmitted remotely to the SI joint to cause pathological changes. The manuscript currently lacks supporting biomechanical data for this transmission.

Response: We thank the reviewer for this valuable feedback. We agree with the concern that the manuscript needed a clearer explanation of how mechanical stress is remotely transmitted from the ankle to cause pathological changes in the left SI joint. While we did not collect direct biomechanical data in this study, we rely on established biomechanical literature to explain this connection. Ikeda et al. (2023) confirmed that short-term cast fixation of a unilateral lower limb causes postural control asymmetry, shifting the center of pressure entirely to the unfixed side. Because the lower limb and pelvis are biomechanically connected, this increased and asymmetric weight-bearing at the ankle transmits abnormal mechanical forces upwards to the SI joint. Supporting this, Shedge et al. (2024) reported that lateral ankle sprains directly lead to SI joint dysfunction. These studies clearly demonstrate the mechanism by which ankle microtrauma causes remote damage to the SI joint. We have added this detailed explanation to the Discussion section (Please refer to lines 238–245 in the revised manuscript).

-Reference-

Ikeda T, Oka S, Tokuhiro J, Suzuki A, Matsuda K. Short-Term Cast Immobilization of a Unilateral Lower Extremity and Physical Inactivity Induce Postural Instability during Standing in Healthy Young Men. Healthcare (Basel). 2023 Sep 13;11(18):2525. doi: 10.3390/healthcare11182525.

Shedge SS, Ramteke SU, Samal S. Integrated Rehabilitation Approach Utilizing Swiss Ball Training, Mulligan Taping, and Mobilization With Movement for Simultaneous Management of Sacroiliac Joint Dysfunction and Lateral Ankle Sprain in a Badminton Athlete: A Case Study. Cureus. 2024 Mar 26;16(3):e56942. doi: 10.7759/cureus.56942.

4. A total of 3 mg of Curdlan was administered in two injections. Please provide references for this specific dosage and discuss whether such a high dose might induce severe systemic arthritis that could potentially mask the localized differences caused by microtrauma.

Response: We sincerely thank the reviewer for raising these important points. Regarding the dosage, the dual injection of 3 mg of Curdlan is an established protocol used to robustly induce ankylosing spondylitis in male SKG mice. This specific regimen has been validated in our previous publications and other studies (Kim et al., 2026; Kim et al., 2023; Jo et al., 2021). We have now added these relevant references to the Methods section (Line 98).

We also acknowledge the concern that Curdlan-induced systemic inflammation might mask the localized response to microtrauma. While Curdlan does provoke systemic responses, including widespread joint and extra-articular inflammation, our data show that it does not mask the microtrauma effect. In mice treated only with Curdlan, inflammation increased equally in both ankles. However, upon applying unilateral immobilization, the inflammatory process and bone destruction were markedly accelerated in the contralateral non-immobilized limb. This clear difference indicates that systemic inflammation does not completely override the local response. Instead, microtrauma acts synergistically as a powerful local aggravating factor, actively promoting disease progression under systemic inflammatory conditions. We have included these findings as Supplementary Figure 3 and added the corresponding description to the revised manuscript(Please refer to lines 182–185 in the revised manuscript).

-Reference-

Jo S, Lee EJ, Nam B, Kang J, Lee S, Youn J, Park YS, Kim YG, Kim TH. Effects of dihydrotestosterone on osteoblast activity in curdlan-administered SKG mice and osteoprogenitor cells in patients with ankylosing spondylitis. Arthritis Res Ther. 2020 May 24;22(1):121. doi: 10.1186/s13075-020-02217-9.

Kim M, Kim K-S, Hong S-J, Youn J, Kim YI and Lee Y-A (2026) Sex-specific COX-2/CREB/ER signaling underlies male susceptibility to pulmonary fibrosis in rheumatoid arthritis-associated interstitial lung disease. Front. Immunol. 17:1753418. doi: 10.3389/fimmu.2026.1753418

Kim YE, Lee JH, Lee EJ, Kim DH, Jeong MR, Hong S, Lee CK, Yoo B, Youn J, Chang EJ, Kim YG. The Expression of the Alpha7 Nicotinic Acetylcholine Receptor and the Effect of Smoking in Curdlan-Administered SKG Mice. Biomedicines. 2023 Oct 11;11(10):2757. doi: 10.3390/biomedicines11102757.

5. Although the authors state that bone mineral density (BMD) remained unchanged, AS is typically characterized by abnormal bone metabolism. It is recommended to provide a detailed table of trabecular bone parameters (e.g., BV/TV, Tb.N, Tb.Th) with specific numerical values rather than relying solely on representative micro-CT images.

Response: We sincerely thank the reviewer for this valuable suggestion. We completely agree that a detailed table of trabecular bone parameters with specific numerical values is necessary to better understand the changes in bone metabolism. As requested, we have added a table detailing these specific numerical parameters. This data is now provided as Supplementary Table 1 in the revised supporting information.

|  | PBS | | Curdlan | |
| --- | --- | --- | --- | --- |
|  | Left  (Non-immobilization) | Right  (Immobilization) | Left  (Non-immobilization) | Right  (Immobilization) |
| BT/TV (%) | 14.60 ± 1.14 | 14.0. ± 1.01 | 8.68 ± 1.22 | 8.73 ± 1.16 |
| Tb.N (1/mm) | 2.38 ± 0.19 | 2.30 ± 0.21 | 1.70 ± 0.19 | 1.69 ± 0.20 |
| Tb.Sp (mm) | 0.026 ± 0.0004 | 0.026 ± 0.0013 | 0.026 ± 0.0018 | 0.025 ± 0.0006 |
| Tb.Th (mm) | 0.061 ± 0.0005 | 0.062 ± 0.0016 | 0.051 ± 0.0024 | 0.051 ± 0.0015 |

6. FGF16 and its expression in muscle are introduced in the Discussion. However, no experimental results for FGF16 are presented in the main Results section. If FGF16 is considered a core finding of this study, these data must be moved from the Supplementary Material to the main body of the manuscript.

Response: We appreciate the reviewer’s thoughtful comment regarding the placement of the FGF16 data. We understand the reviewer's point that core findings should be presented in the main text. However, we currently view the FGF16 data as an intriguing preliminary observation rather than a fully validated "core" finding. Importantly, the initial RNA-sequencing analysis used to identify the upregulation of FGF16 was conducted with a limited sample size (n=1 per group) strictly as an exploratory screening tool. Although we subsequently supported this observation with mRNA analysis and IHC, our current study lacks both the statistical robustness from the sequencing data and sufficient functional validation regarding how FGF16 actively contributes to the disease pathology. To avoid overstating our conclusions based on this insufficient sample size and the lack of mechanistic depth, we believe it is scientifically more appropriate to retain these observational data in the Supplementary Material. We have transparently discussed these limitations in the Discussion section, positioning FGF16 as a potential target for future investigation.

7. The hypothesis that FGF16 promotes inflammation is currently based only on RNA-seq inference. To support this claim, the authors should supplement this with in vitro experiments (e.g., FGF16 stimulation of synovial cells) or in vivo blocking/intervention experiments.

Response: We appreciate the reviewer’s constructive feedback regarding the need for functional validation of FGF16.To investigate its potential role, we actively performed comprehensive in vitro assays. While the reviewer suggested using synovial cells, we focused on primary target cells directly involved in AS pathology and treated LPS-stimulated macrophages, osteoblasts, and osteoclasts with FGF16 (10, 25, and 50 ng/mL). However, we did not observe any significant direct phenotypic changes or modulatory effects in these cell types under our experimental conditions(please refer to author response figure 1-4). Crucially, we would like to clarify that our initial RNA-sequencing data which originally highlighted FGF16 was generated as an exploratory screening tool with a limited sample size (n=1 per group). Given this insufficient sample size and the subsequent lack of direct response in key bone and macrophage lineages, we recognized the risk of overstating our conclusions. Consequently, we intentionally refrained from moving these preliminary findings to the main text or performing extensive in vivo intervention studies at this stage. Instead, as recommended, we have toned down our claims and transparently discussed these mechanistic limitations in the Discussion section (Lines 269–282). We have also raised the alternative possibility that FGF16 might instead modulate adaptive immune cells, such as Th1 or Th17 subsets, which warrants further investigation in future studies.

8. Increased MMP13 expression was observed in the SI joint. The authors should delve deeper into how microtrauma induces the overexpression of matrix metalloproteinases—is it a result of the inflammatory cytokine cascade or direct mechanical signaling transduction?

Response: We thank the reviewer for this excellent suggestion. We propose that the increased MMP13 expression observed in the SI joint is mediated via mechanical signaling transduction. In our study, a significant difference was observed specifically in the non-immobilized (left) SI joint within the Curdlan-injected cohort (Figures 3A and 3B). This indicates that the direct effect of Curdlan-induced systemic inflammatory cytokines on the SI joint is relatively minor under our experimental conditions, and that the pathological elevation of MMP13 is predominantly triggered by compensatory mechanical stress. This rationale is strongly supported by previous literature. Zhou et al. (2024) showed that MMP13 expression increases significantly following mechanical joint injury in post-traumatic osteoarthritis models. Additionally, Bedingfield et al. (2021) demonstrated that mechanical loading directly upregulates MMP13 expression in mice. Therefore, we conclude that direct mechanical signaling transduction is the main driver of MMP13 overexpression in the affected SI joint.

-Reference-

Zhou D, Wei Y, Sheng S, Wang M, Lv J, Zhao B, Chen X, Xu K, Bai L, Wu Y, Song P, Cao L, Zhou F, Zhang H, Shi Z, Su J. MMP13-targeted siRNA-loaded micelles for diagnosis and treatment of posttraumatic osteoarthritis. Bioact Mater. 2024 Apr 23;37:378-392. doi: 10.1016/j.bioactmat.2024.04.010.

Bedingfield SK, Colazo JM, Yu F, Liu DD, Jackson MA, Himmel LE, Cho H, Crofford LJ, Hasty KA, Duvall CL. Amelioration of post-traumatic osteoarthritis via nanoparticle depots delivering small interfering RNA to damaged cartilage. Nat Biomed Eng. 2021 Sep;5(9):1069-1083. doi: 10.1038/s41551-021-00780-3.

9. A hallmark of AS is pathological new bone formation. While joint destruction was observed, the evidence for "ankylosis" (new bone formation) remains relatively weak. It is suggested to extend the observation period to capture potential ectopic ossification or enthesophyte formation.

Response: We thank the reviewer for this valuable suggestion. As demonstrated in our previous study (Jo et al., 2023), ectopic bone formation induced by inflammation becomes evident in the ankles of Curdlan-treated SKG mice at more than 4 weeks post-injection. Therefore, the total experimental period in the present study was set to 5 weeks to adequately capture this pathology. As expected, our micro-CT analysis revealed the onset of inflammation-associated new bone formation in the ankle joints during this period. However, we fully agree with the reviewer's comment that a longer observation period could provide deeper insights into disease progression and advanced ankylosis. Accordingly, we have noted this in the manuscript and plan to extend the experimental duration in our future studies to further evaluate the long-term effects on ectopic bone formation.

-Reference-

Jo S, Lee SH, Park J, Nam B, Kim H, Youn J, Lee S, Kim TJ, Sung IH, Choi SH, Park YS, Inman RD, Kim TH. Platelet-Derived Growth Factor B Is a Key Element in the Pathological Bone Formation of Ankylosing Spondylitis. J Bone Miner Res. 2023 Feb;38(2):300-312. doi: 10.1002/jbmr.4751. Epub 2022 Dec 9.

10. The reference list needs to be updated. Please ensure that a significant majority of the cited literature consists of high-quality studies published within the last three years to reflect the current state of the field.

Response: We appreciate the reviewer’s valuable recommendation regarding our reference list. We completely agree that the cited literature must accurately reflect the most current state of the field. In accordance with your constructive suggestion, we have thoroughly reviewed and updated our references. We have removed outdated citations and explicitly incorporated a significant number of high-quality studies published within the last three years. This comprehensive update ensures that our manuscript aligns well with the latest trends and recent advancements in the field.

11. While the authors acknowledge the lack of FGF16 intervention experiments in the Discussion, a more rigorous and sincere discussion regarding the clinical translational significance and the limitations of the current animal model is encouraged.

Response: We thank the reviewer for this excellent suggestion. We acknowledge that our previous discussion regarding the limitations of the FGF16 research was insufficient, particularly concerning its clinical applicability. Following your advice, we have re-evaluated our conclusions and incorporated a more rigorous discussion on the limitations of the current animal model and the clinical translational significance of our findings. We have moderated our claims to cautiously suggest that FGF16 may act as a contributing factor to joint inflammation following microtrauma, rather than drawing definitive clinical conclusions. These sincere revisions have been added to the Discussion section (Please refer to lines 262–264 in the revised manuscript).
