# Supplementary material for: Effects of microtrauma on joint structural changes in the Curdlan-injected SKG mouse model of ankylosing spondylitis
Source: PLoS One. 2026 Oct 15;21(10):e0360912. doi: 10.1371/journal.pone.0360912 (PMC13671563; doi:10.1371/journal.pone.0360912)
Supplement: Supplementary file 3 [file pone.0360912.s005.docx]

**Response to reviewer #3 comments**

(indicated in blue)

Reviewer #3:

1. The authors established mechanical overload on the unimmobilized side by immobilizing the contralateral hind limb to induce microtrauma. However, it would be clearer if the authors could clarify whether the total physical activity/locomotion of the mice was monitored after unilateral immobilization. Please briefly discuss in the Discussion section whether reduced total movement or altered gait kinematics could confound the system inflammation or mechanical stress distribution on the non-immobilized side.

Response: We thank the reviewer for this excellent suggestion. We acknowledge that our previous discussion regarding the limitations of the monitors in physical activity/locomotion research was insufficient, particularly concerning their system inflammation or mechanical stress distribution. Previous studies have demonstrated that unilateral joint immobilization or restriction can alter walking speed and gait mechanics and induce compensatory changes in mechanical loading at non-immobilized and contralateral sites (Lage et al., 1995; Walk et al., 2024). Moreover, reduced locomotor activity and mechanical loading can influence musculoskeletal homeostasis including bone remodeling and maintenance (Liphardt et al., 2020). Therefore, some changes observed at non-immobilized sites in our study may reflect secondary effects of reduced overall physical activity and redistributed mechanical loading due to altered gait, rather than site-specific responses alone. Future studies incorporating quantitative assessments of locomotor activity and gait biomechanics will be necessary to distinguish these systemic effects from local responses. We added this discussion to the revised manuscript (Please refer to lines 241–254).

-Reference-

Lage KJ, White SC, Yack HJ. The effects of unilateral knee immobilization on lower extremity gait mechanics. Med Sci Sports Exerc. 1995 Jan;27(1):8-14.

Walker J, Thomas A, Stolycia ML, Wilkins RA, Lunn DE. Acute biomechanical responses to wearing a controlled ankle motion (CAM) Walker boot during walking. Gait Posture. 2024;108:208–214. doi:10.1016/j.gaitpost.2023.12.011.

Liphardt AM, Windahl SH, Sehic E, Hannemann N, Gustafsson KL, Bozec A, Schett G, Engdahl C. Changes in mechanical loading affect arthritis-induced bone loss in mice. Bone. 2020 Feb;131:115149. doi: 10.1016/j.bone.2019.115149. Epub 2019 Nov 9.

2. The manuscript clearly shows that microtrauma exacerbates synovial inflammation and enthesitis with elevated TNF-α, IL-1β, and IL-6. To enrich the Discussion regarding how local biomechanical stress alters the joint microenvironment and inflammatory macrophage infiltration, the authors are encouraged to elaborate on the crosstalk between physical injury and immune microenvironment biomarkers. Citing recent integrative studies on synovial inflammation and immune cell polarization biomarkers in arthritis (J Multidiscip Healthc, 2025; PMID: 40765736) would provide a broader translational context.

Response: We thank the reviewer for this valuable feedback. Mechanical stress and microtrauma may contribute to inflammation not only through direct tissue damage but also by altering the local immune microenvironment. Tissue injury can promote the release of inflammatory mediators and facilitate the recruitment and activation of immune cells, including macrophages, thereby amplifying local inflammatory responses. Macrophage polarization is increasingly recognized as an important component of the inflammatory joint microenvironment. Recent integrative analyses of osteoarthritis synovial tissues have identified macrophage polarization-related molecular signatures (such as MYC, SIK1, NFIL3) associated with immune cell infiltration, further supporting a close relationship between synovial inflammation and macrophage phenotypes [He et al., 2025]. In this context, the increased expression of TNF-α, IL-1β, and IL-6 observed following microtrauma in our study may reflect, at least in part, alterations in the local immune microenvironment induced by biomechanical stress. Further studies characterizing macrophage infiltration and polarization will be required to clarify the cellular mechanisms linking microtrauma to local inflammation. We added this discussion to the revised manuscript (Please refer to lines 277–290).

-Reference-

He Q, Liu L, Hu X, Lin L, Song Z, Xia Y, Lin Q, Wei J, Li S. Integrated Bioinformatics and Experimental Validation Reveal Macrophage Polarization-Related Biomarkers for Osteoarthritis Diagnosis. J Multidiscip Healthc. 2025 Aug 1;18:4589-4612. doi: 10.2147/JMDH.S537507. eCollection 2025.

3. In Figures 3 and 5, micro-CT analysis was performed to evaluate bone density and ectopic bone formation. Could the authors specify the exact ROI selection criteria for the enthesis versus the joint space in the methodology? Additionally, please briefly discuss the balance between osteoclasts and osteoblasts under mechanical strain, as mechanical stress often drives dual effects (bone resorption vs. new bone formation) in ankylosing spondylitis.

Response: We thank the reviewer for this valuable feedback. We have clarified the anatomical definition of the ROI used for micro-CT analysis in the Methods section. Briefly, femoral BMD was assessed using a standardized ROI in the distal femoral trabecular region, whereas ectopic new bone formation in the ankle was evaluated using an ROI centered on the entheseal/peri-entheseal region rather than the joint space. The same anatomical landmarks and ROI criteria were consistently applied across all experimental groups. We have added this detailed explanation to the Methods section (Please refer to lines 120–125 in the revised manuscript).

We also agree that mechanical stress may differentially affect bone resorption and new bone formation in AS. We have therefore added a brief discussion regarding the balance between osteoclast- and osteoblast-mediated bone remodeling under inflammatory and mechanical conditions. Mechanical stress may also exert distinct and context-dependent effects on bone remodeling in AS. In an inflammatory environment, pro-inflammatory cytokines can promote osteoclast differentiation and activity, whereas repetitive mechanical stress, particularly at entheseal sites, may promote local osteogenic responses and pathological new bone formation [Nakamura A et al., 2024; Jacques P, et al., 2014]. Thus, bone resorption and pathological new bone formation are not necessarily mutually exclusive but may represent spatially and temporally distinct aspects of dysregulated bone remodeling in AS [Nakamura A et al., 2024]. Notably, femoral BMD was not significantly altered in our study, whereas ectopic bone formation at the ankle was affected. These findings suggest that the effect of mechanical loading in our model may be more pronounced in local pathological bone formation than in generalized changes in bone mass. However, because BMD alone does not directly reflect osteoclast or osteoblast activity, further studies evaluating these cell populations at mechanically stressed sites will be required. We added this discussion to the revised manuscript (Please refer to lines 241–254).

-Reference-

Nakamura A, Towheed T. Pathogenesis, assessment, and management of bone loss in axial spondyloarthritis. Semin Arthritis Rheum. 2024 Feb;64:152345. doi: 10.1016/j.semarthrit.2023.152345. Epub 2023 Dec 12.

Jacques P, Lambrecht S, Verheugen E, Pauwels E, Kollias G, Armaka M, Verhoye M, Van der Linden A, Achten R, Lories RJ, Elewaut D. Proof of concept: enthesitis and new bone formation in spondyloarthritis are driven by mechanical strain and stromal cells. Ann Rheum Dis. 2014 Feb;73(2):437-45. doi: 10.1136/annrheumdis-2013-203643. Epub 2013 Aug 6.

4. The discussion on signaling pathways involved in microtrauma-induced joint destruction could be further deepened. Environmental and physical stresses often trigger inflammatory cascades through interconnected biological networks. Incorporating relevant literature on network-level target identification and physical/environmental stress response in joint pathology (BMC Pharmacol Toxicol, 2026; PMID: 41742320; Comb Chem High Throughput Screen, 2026; PMID: 42483912) would enhance the theoretical framework of the manuscript.

Response: We sincerely thank the reviewer for this important suggestion. We agree that microtrauma-induced joint pathology may involve coordinated interactions among multiple signaling pathways rather than a single molecular pathway. Accordingly, we have expanded the Discussion to address the potential involvement of interconnected signaling networks in the response to physical and environmental stress. In particular, citing the suggested studies (He et al., 2026; Wang et al., 2026), we have added that several signaling pathways, including estrogen, thyroid hormone, ErbB, MAPK, PI3K-Akt, and mTOR signaling, may be involved. We added this discussion to the revised manuscript (Please refer to lines 291–298).

-Reference-

He Q, Li S, Chen Y, Liu Y, Li Y, Min S, Yi S, Li F, Zhou X. Investigating the mechanisms by which bisphenol A affects osteoarthritis through a novel network toxicology framework and experimental validation. BMC Pharmacol Toxicol. 2026 Feb 25;27(1):50. doi: 10.1186/s40360-026-01108-0.

Wang W, He Q, Zhang Z, Zhang T, Xiong Z, Zhou T, Feng R, Liu X. Exploring the Potential Mechanism of Isoliquiritigenin in the Treatment of Osteoarthritis through Integrated Network Pharmacology, Molecular Docking, and Experimental Validation. Comb Chem High Throughput Screen. 2026 Jul 14. doi: 10.2174/0113862073486376260621183559.
